# Interaction of disparity size and depth structure on perceived numerosity in a three-dimensional space

**DOI:** 10.1371/journal.pone.0230847

**Published:** 2020-04-02

**Authors:** Saori Aida, Yusuke Matsuda, Koichi Shimono

**Affiliations:** 1 Department of Computer Science, Tokyo University of Technology, Hachioji, Tokyo, Japan; 2 Department of Logistics and Information Engineering, Tokyo University of Marine Science and Technology, Koto, Tokyo, Japan; University of Trento, ITALY

## Abstract

The number of elements in two stereo-surfaces parallelly overlapped in depth is overestimated compared to that in a single flat surface, even when both have the same number of elements. Using stereoscopic pairs of elements, we evaluated two hypotheses on the overestimation: one that a higher-order process, forming a background surface, increases the number of perceived elements, and the other that the number of elements potentially occluded by the elements on a front surface is taken accounted for. The data from four experiments showed that (a) when binocular disparity between (or among) stereoscopic elements was small, the overestimation occurred for the stimuli we used—a two-surface-overlapping stimulus, where the likelihood for the process to operate was manipulated by changing the averaged luminance of each surface, a volumetric stimulus, where the likelihood for the background surface to be formed would decrease, and a two-non-overlapping-surface stimulus, where the surfaces in depth were not overlapped—, and (b) when binocular disparity was large, the overestimation occurred for the two-surfaces-overlapping stimulus, when the averaged luminance of the two surfaces were the same, and for the volumetric stimulus, but diminished for the surface-overlapping stimulus, when the averaged luminance differed between the surfaces and for the surfaces-non-overlapping stimulus. These results cannot be explained either hypothesis only. We explain the results by postulating that the sensory system processing disparities of elements interferes with that estimating the number of elements, resulting in an overestimation of the elements in a stereo-stimulus, and the disparity range within which the interference occurs may depend on the stimulus depth structure.

## Introduction

Humans are capable of determining the number of objects that are scattered in front of them rather accurately. This capability is referred to as numerosity perception. Previous studies have hypothesized that the process of the numerosity perception comprises three sub-processes: subitizing, estimation, and counting [1, 2]. Subitizing is the rapid enumeration of six or fewer objects. Estimation is an instantaneous determination of the numerosity of six or more objects without counting individual objects. Counting, as the term suggests, signifies the process of counting objects either individually or in units of two or three. Since Kaufman (1949) [1] proposed the hypothesis, several studies have been conducted to clarify the characteristics of these sub-processes, with recent studies identifying the brain regions that mediate the respective sub-processes (e.g., [3–5]).

Of these sub-processes of numerosity perception [6–9], this study focused on the process of numerical estimation. Conventionally, the process of numerical estimation has primarily been performed based on the ability of an individual to estimate the number of elements presented on a flat surface (e.g., [10, 11]) or the capability to compare the quantities of two consecutively or simultaneously presented elements (e.g., [12, 13]). However, most researches on the numerosity perception are limited for the elements that are arranged in a two-dimensional (2-D) plane but not in a three-dimensional (3-D) space. Given that the numerosity perception has developed in the course of phyletic evolution to process objects in a 3-D space efficiently, the numerosity perception should be examined with the elements arranged in a 3-D space as well as with those in a 2-D space; we believe that the arrangement of elements in a 3-D space is more ecologically relevant than that in a 2-D space to reveal the process of numerosity estimation.

In line with the above discussion, several studies have presented elements in a 3-D space to investigate the numerosity judgments [14–17]. For instance, Aida, Kusano, Shimono, & Tam (2015) [18] employed a 3-D stimulus in which elements were perceived on two parallel, overlapping, transparent, stereoscopic surfaces (POTS). They compared the number of elements in a two-POTS stimulus with that of elements in a 2-D stimulus with zero disparity (a single-surface stimulus). They found that the number of elements, as represented by pixelated dots, was perceived to be higher in the overlapping-surface stimulus than in the single surface stimulus even when their physical numbers were identical to each other. This finding shows that the number of elements of a 3-D stimulus is overestimated, compared to that of a 2-D stimulus, suggesting that a process(es) for estimating the number of elements in a 2-D space and for in a 3-D space inherently differs, corroborating our argument that it is necessary to use a 3-D stimulus to understand the numerical estimation fully.

Two hypotheses have been proposed to explain the overestimation of the number of 3-D elements (or 3-D overestimation phenomenon): the back-surface-bias hypothesis [14, 17, 19] and the occlusion hypothesis [14, 17]. The back-surface-bias hypothesis postulates that when elements seen behind in a two-POTS stimulus are perceived as a background surface, the apparent density (or number) of the constituent elements on the background surface increases [19], resulting in a 3-D overestimation phenomenon [17]. According to this hypothesis, the visual system relies on the density of elements on the back surface. Meanwhile, the occlusion hypothesis postulates that the visual system factors in the possibility that the elements perceived in the front surface can occlude elements behind and adds the number of the occluded elements and physical elements, resulting in a 3-D overestimation phenomenon [14]. According to this hypothesis, the visual system relies on the number of “hidden elements” for numerical estimation.

However, the hypotheses have not been tested directly yet, although previous results are generally consistent with either hypothesis. Schütz (2012) [17] and Aida et al. (2015) [14], for example, examined the perceived number of elements on a front surface and a back surface of a two-POTS stimulus separately; they kept the number of total elements on the stimulus constant while manipulating the ratio of the number of elements on a front surface to that on a back surface. While Schütz [17], but not Aida et al. [14], found the underestimation of the number for a front surface, both found the overestimation of the number for a back surface. The overestimation for a back surface is consistent with the back-surface-bias hypothesis if the visual system regards a back surface of a two-POTS stimulus as a background [14, 17]. The occlusion hypothesis can also explain the overestimation, if it can be “interpreted as an overcompensation of occlusion” [17] (*p*. 12).

In the present study, we performed four experiments to evaluate the predictive performance of the back-surface-bias and occlusion hypotheses. The first hypothesis predicts that if constituent elements are less likely to be perceived as a background surface, a 3-D overestimation phenomenon would be less likely to occur. The second hypothesis predicts that when there is a lack of overlap between a front and a back surface, there would be no hidden elements, and thus, a 3-D overestimation phenomenon would not occur. Experiments 1 and 2 investigated the predictions from the first hypothesis, and Experiments 3 and 4 explored the predictions from the second hypothesis. In all experiments, observers were given a task to compare the numbers of elements in 2-D and 3-D stimuli, which were presented side-by-side.

## General methods

### Experimental devices

Stimuli were produced using MATLAB software running on Windows computers in each experiment. The computers were equipped with an OptiPlex 9020 system (DELL) for the smaller disparity condition and an FMV ESPRIMO WD2/A3 system (FUJITSU) for the larger disparity condition. Stimuli were presented on two monitors: Diamondcrysta RDT198LM (MITSUBISHI) for the smaller disparity condition and CS230-CN (EIZO) for the larger disparity condition. The spatial resolution of the monitors was 1280 × 1024 pixels. Stimuli were presented with a stereoscope fitted with two perpendicular mirrors. Further, the centers of the stimuli were at the height of 107 cm from the floor, and the distance from observers was set at 60 cm. Observers were asked to sit on a chair set at the height of 40 cm from the floor, and their heads were fixed on a chin support.

### Stimuli

**Fig 1:**
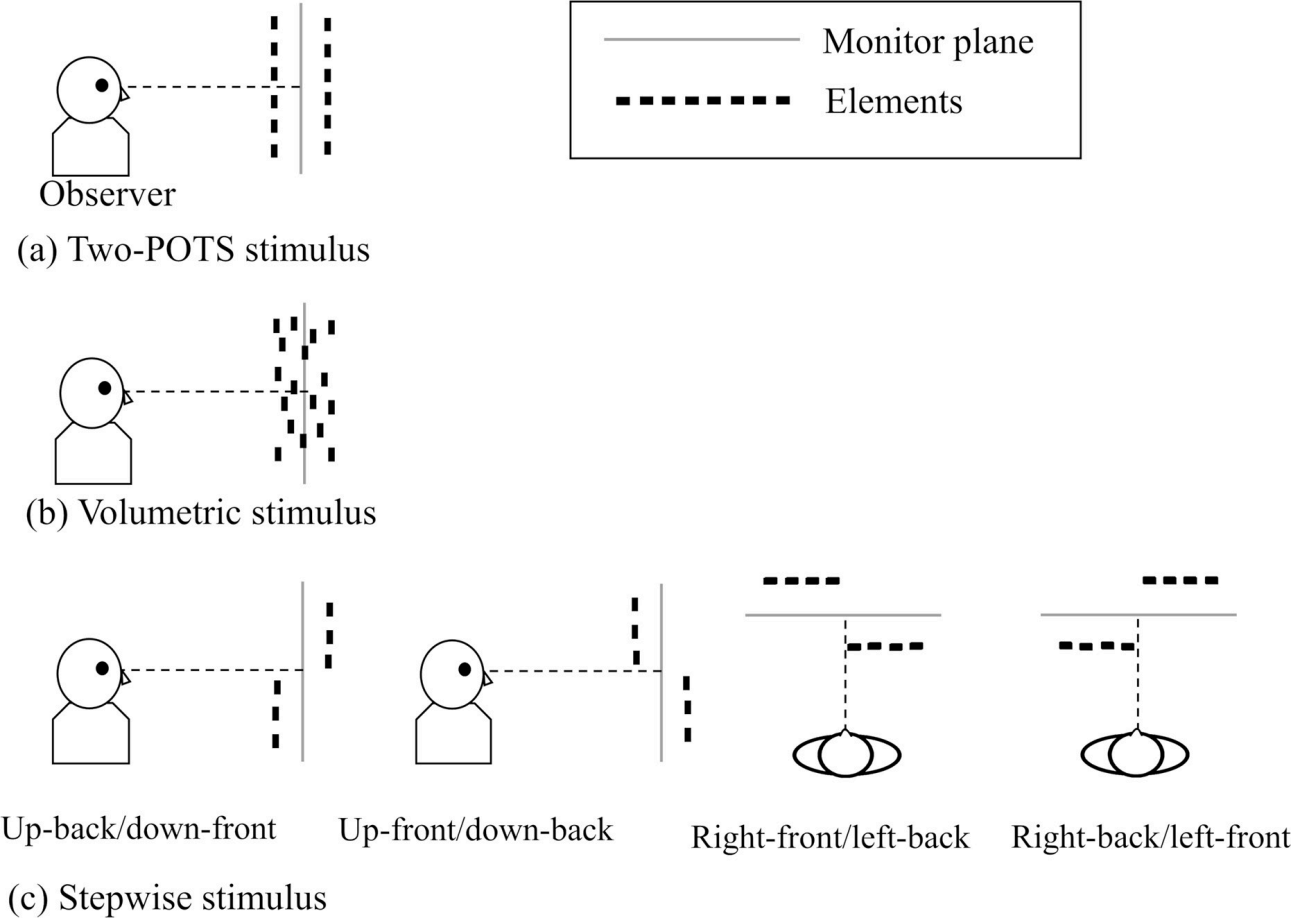
Schematic illustrations of 3-D stimuli included in the experiments. (a) a two -POTS stimulus, (b) a volumetric stimulus, and (c) a stepwise stimulus (up-back/down-front, up-front/down-back, right-front/left-back, or right-back/left-front stimulus) (from left to right). Illustrations were drawn from the side view except for those of right-front/left-back and left-front/right-back stimuli, which were drawn from the top view. Gray lines illustrate the monitor planes, and the solid black rectangular boxes represent the position of the elements. See the text for a detailed description.

The stimuli included a 2-D stimulus and a 3-D stimulus, both of which were presented in the form of a random-dot stereogram (RDS). The 2-D stimulus, when fused, would appear as a single flat surface on the monitor plane. There were three different types of 3-D stimulus: when fused, it appeared as two-POTS, one of which was in front of and the other behind the monitor plane (see Fig 1A); as a "volume," which was expected not to constitute surfaces (see Fig 1B); or as two non-overlapping surfaces or stepwise surfaces. One of the stepwise surfaces would be seen in front of and the other behind the monitor plane, but they were horizontally or vertically separated and did not overlap (see Fig 1C). The size of the 2-D and 3-D stimuli was 8.6° × 8.6° (9.0 cm × 9.0 cm) in all the experiments except for that of the 2-D stimulus, which was 4.3°× 8.6° (4.5 cm × 9 cm) in Experiment 4. In Experiment 3, the size of the upper or lower half of the stepwise stimulus was 4.3° × 8.6° (4. 5 cm x 9.0 cm) and the size of its right or left half was 8.6° × 4.3° (9.0 cm x 4.5 cm). The 2-D and 3-D stimuli were used as the standard and comparison stimuli, respectively, in each experiment. A fixation was a cross symbol, which was placed between the center of the two stimuli. The cross symbol had zero disparity with respect to the monitor.

The stimuli consisted of rectangular elements, each of which had a size of 5.7 × 5.7 arcmin. The elements were white and/or black and presented on a gray background whose size was 22.3° × 38.6° for the smaller disparity condition and 26.8° × 45.9° for the larger disparity condition. The luminances of white and black elements were set at 0.13 and 30.85 cd/m^2^, respectively. The luminance of a gray background was set at 12.93 cd/m^2^. The luminance was measured with a luminance photometer (LS100, Konica Minolta). The elements in a 2-D stimulus and the background had zero disparity with respect to the monitor plane, and those of a two-POTS and a stepwise stimulus had 6.8 or 12.7 arcmin total disparity. With respect to the monitor plane, the elements in the front and back surfaces had +3.4 and -3.4 arcmin disparity, respectively, for the stimulus with 6.8 arcmin total disparity, and +6.4 arcmin and -6.4 arcmin disparity, respectively, for the stimulus with 12.7 arcmin total disparity. The elements of a volumetric stimulus had five different disparities, and their total disparity was 6.8 or 12.7 arcmin. With respect to the monitor plane, the outermost elements had +3.4 and -3.4 arcmin disparities, the middle elements had zero disparity, and the innermost elements had +1.7 and -1.7 arcmin disparity for the stimulus with 6.8 arcmin total disparity. With respect to the monitor plane, the outermost elements had +6.4 and -6.4 arcmin disparities, the middle elements had zero disparity, and the innermost elements had +3.4 and -3.4 arcmin disparity for the stimulus with 12.7 arcmin total disparity. Positive and negative signs represented crossed and uncrossed disparities, respectively. When the elements with crossed disparities were fused, they were perceived in front of the monitor plane; when those with uncrossed disparities were fused, they were perceived at the behind of the monitor plane; and when the background was fused, they were perceived at the monitor plane. The positions of elements with the same binocular disparity in all the stimuli were randomly assigned and manipulated not to overlap or contact to adjacent elements. The positions of the 3-D stimulus elements were also manipulated to ensure binocular fusion (see [18, 20, 21]).

### Procedure

For each trial in each experiment, observers were asked to select which one of the two stimuli (2-D comparison and 3-D standard) appeared to contain more elements than the other or to perform the two-alternative forced-choice task. The observers performed the task at their own pace for the experimental trials, during which they were instructed to fixate the gaze point and allowed to take breaks whenever they felt tired.

### Observers

Twenty-one observers participated in this study: 7 observers (7 males), whose ages were ranging from 22 to 23, in Experiment 1 and 14 observers (11 males), whose ages were ranging from 22 to 28, in Experiments 2 to 4. In Experiments 2–4, the same 7 out of 14 observers were randomly assigned to the smaller-disparity condition, and the remaining was assigned to the larger-disparity condition, and the lead author of this study (female) was one of the 14 observers. The visual acuity of the observers, either aided or unaided, was normal. The results of a stereo fly test (STEREO OPTICAL) demonstrated that all observers could perform stereopsis for disparities of at least 1.0 arcmin. All observers except the lead author were not aware of the aims of the study. Observers gave informed consent prior to taking part in the experiments, which were conducted in accordance with the ethical principles embedded in the Declaration of Helsinki. The protocol was approved by the institutional review boards at the Tokyo University of Technology.

### Psychophysical data analysis

**Fig 2:**
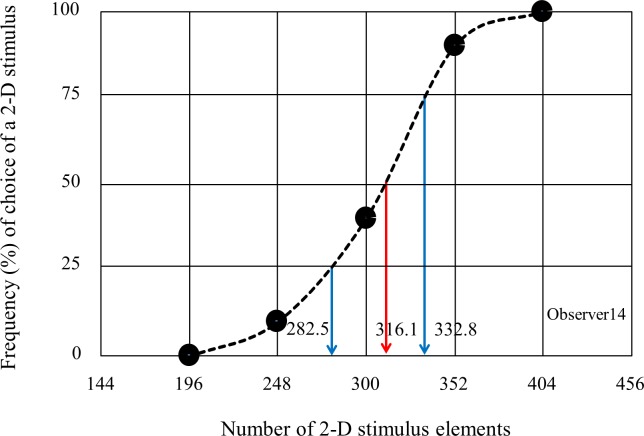
Schematic explanation of how to calculate the PSE and JND. The percentage (%) (*y*-axis) of responses that the number of 2-D stimulus elements was judged to be higher than that of 3-D stimulus elements was plotted against the number of 2-D stimulus elements (*x*-axis). The x-axis value yielding a 50% response in the fitted psychometric function represents the PSE of the 2-D stimulus. Half of the difference between the x-axis values yielding a 25% and a 75% response in the function represents the JND. For instance, when the number of right-front/left-back stimulus elements was fixed at 300, the number of 2-D stimulus elements varied at five levels from 196–404. In this case, PSE and JND of the observer14 were determined to be 316.1 and 25.2, respectively. The red vertical arrow shows the point producing a 50% response in the fitted function. The left and right blue vertical arrows show the points producing a 25% and a 75% response, respectively, in the fitted function.

From the data in each experiment, we calculated the point of subjective equality (PSE) and the just noticeable difference (JND). PSE was defined as the number of 2-D stimulus elements perceived to be the same as that of 3-D stimulus elements. JND was defined as the minimum difference between the number of 2-D stimulus elements and 3-D stimulus elements that can be reliably discriminated 50% of the time. They were extracted from a psychometric function fitted to the percentage of the responses in which the number of 2-D stimulus elements was reported to be higher than that of 3-D stimulus elements, against the number of 2-D stimulus elements. The method used to calculate the PSE and JND is illustrated in Fig 2, which plots one observer's (observer14) responses to the stepwise 3-D stimulus (right-front/left-back) in Experiment 3. The psychometric function fitted was a logistic function as in Aida et al. (2015) [18]. The number of 2-D stimulus elements that produced a 50% response in the psychometric function was determined to be the PSE. We subtracted the number of 3-D stimulus elements from the PSE to determine a bias of the PSE. We judged that when the bias was positive, overestimation in numerical judgment had occurred, and when negative, underestimation had occurred. Furthermore, half of the difference in the number of 2-D stimulus elements between 25% and 75% points in the psychometric function was determined to be the JND. We regarded the JND as an index to show the task difficulty; as the task increased, the JND would increase. We determined the PSE and the JND for each observer and each condition. In this study, the presented position of a 2-D stimulus (right or left of the mid-sagittal plane on the monitor) was not regarded as a variable.

## Experiment 1

We evaluated the back-surface-bias hypothesis that holds that the back surface of the two-POTS stimulus affects the numerosity judgment through a higher-order process that assigns the binocular disparity of the elements in the surface to their surrounding blank areas [14, 17, 19]. According to the hypothesis, the higher-order process is likely to operate when the surface is considered to be an opaque background [19]. In this experiment, we manipulated the likelihood by introducing a gray surface between the two stereo-surfaces and changing the average luminance of each surface. Our logic for the manipulation was from a daily life observation that a gray translucent surface operates to attenuate the luminance of a background surface. We assumed that the higher-order process has developed in the course of phyletic evolution (e.g., [19]) and, thus, is more likely to operate when a two-POTS stimulus is viewed under an ecologically valid condition than an invalid condition. We further assumed that when a back surface with black elements (surface with lower luminance) was viewed through a middle gray surface (with middle luminance) and a front surface with white elements (surface with higher luminance), it is ecologically valid, but when the order of the black and white elements was reversed with respect to the gray surface, it was ecologically invalid. Consequently, the overestimation of elements would occur for two-POTS stimuli with front-white/back-black surfaces but not for that with front-black/back-white surfaces.

### Method

#### Stimuli

**Fig 3:**
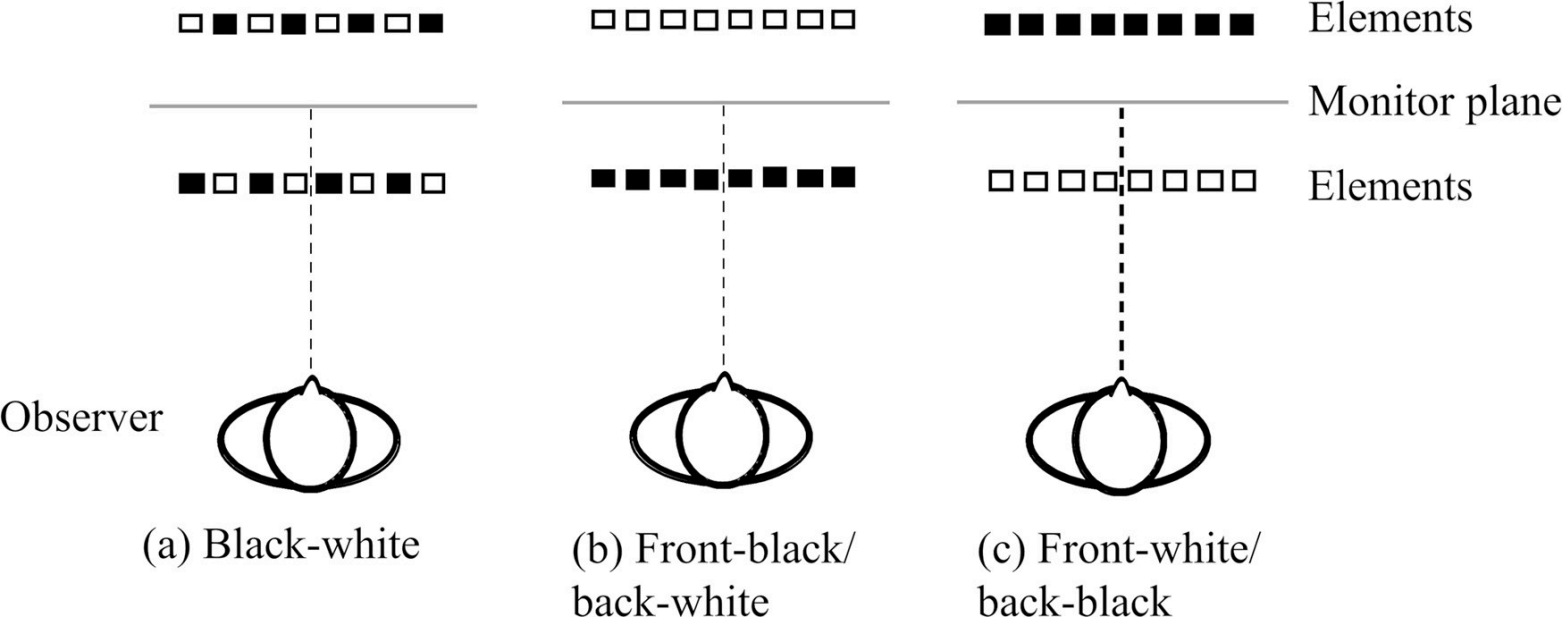
Schematic illustrations of three different two-POTS stimuli used in Experiment 1. (a) black-white, (b) front-black/back-white and (c) front-white/back-black two-POTS stimuli (from left to right). Illustrations were drawn from the top view. While, for descriptive purpose, black and white elements were depicted as if they were placed alternatively for the black-white stimulus, their locations were randomly determined in the experiment.

The 3-D standards stimuli were three different two-POTS stimuli: black-white, front-black/back-white, and front-white/back-black stimuli (see Fig 3). For the black-white two-POTS stimulus, the color of the half of the elements was black and that of the other half was white in each surface (Fig 3A). For the front-black/back-white two-POTS stimulus, the colors of elements on the front and back surfaces were black and white, respectively (Fig 3B). For the front-white/back-black two-POTS stimulus, the colors of elements on the front and back surfaces were white and black, respectively (Fig 3C). The color of the middle surface was gray. The total number of elements on each of three two-POTS stimuli was 300, and each of the front and back surfaces had one half (150). In the black-white two-POTS stimulus, the number of white and black elements was 75 each on each surface. The numbers of simultaneously presented 2-D stimulus elements varied at five levels with 300 set as the baseline. The initial values and ranges of the step sizes for the five levels for the 2-D comparison was varied among different observers; we adjusted the ranges to match the responses necessary to fit the psychometric function for each observer. The initial values ranged from 180 to 196, and the step sizes ranged from 52 to 60.

#### Procedure

The experiment comprised three blocks, each for one of the three different 3-D (standard) stimuli: black-white, front-black/back-white and front-white/back-black two-POTS stimuli. The order of the block was randomized among observers. In each block, the location of the 3-D standard (left or right of the midsagittal plane), size of the binocular disparity (6.8 and 12.7 arcmin), and the number of 2-D (comparison) stimulus elements were randomly selected with five repetitions. Consequently, each observer was presented with stimuli for a total of 300 times (3 standards × 2 binocular disparities × 5 numbers of elements on the comparison × 2 locations × 5 repetitions).

### Results and discussion

**Fig 4:**
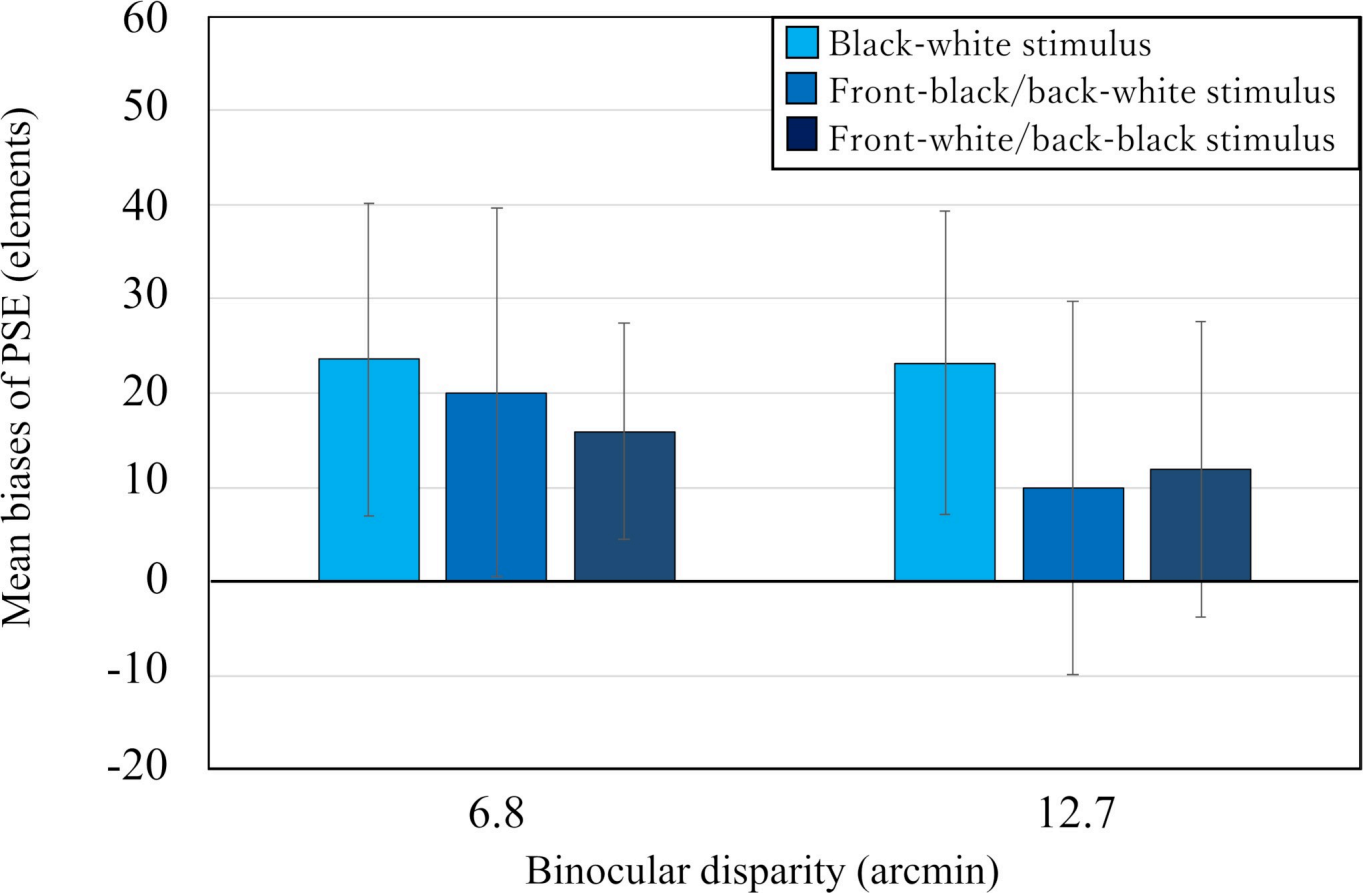
Results from Experiment 1. Mean biases of PSE for the three types of two-POTS stimuli. The vertical and horizontal axes represent the mean biases of PSE and binocular disparity, respectively. Error bars indicate 95% confidence interval.

First, we conducted a two-way repeated measures ANOVA (3 two-POTS stimuli × 2 binocular disparities) on the bias of the PSE. The analysis showed that the main effect of the stimulus, *F* (2, 12) = 2.87, *p* = 0.10, and that of binocular disparity, *F* (1, 6) = 0.61, *p* = 0.46, and their interaction, *F* (2, 12) = 1.08, *p* = 0.37, were not statistically significant. Fig 4 showed the mean biases separately for each two-POTS stimulus with the binocular disparity as a parameter with the error bars indicating 95% confidence interval (95% CI). As can be seen in Fig 4, the mean biases were nearly the same, and the 95% CIs overlapped among the three POTS stimuli in each disparity condition, being consistent with the result of ANOVA.

Next, we analyzed the 95% CI for the mean bias of each condition as an index of the overestimation phenomenon; when the limit was larger than zero, we regarded that the overestimation of the number of elements on the two-POTS stimulus had occurred. As can be seen in Fig 4, the lower limit is larger than zero when the binocular disparity is small (95% CIs for the black-white, front-black/back-white, and front-white/back-black two-POTS stimuli were 23.6 ± 16.6, 20.1 ± 19.6, and 15.9 ± 11.4, respectively). On the contrary, when the disparity was large, the lower limit was larger than zero only for the black/white two-POTS stimulus and smaller than zero for the other two-POTS stimuli (95% CIs for the black-white, front-black/back-white, and front-white/back-black surfaces stimuli were 23.2 ± 16.1, 9.9 ± 19.8, and 11.8 ± 15.7, respectively). These results indicated that it depended on the disparity size of the two-POTS stimulus and the difference of the averaged luminance between the surfaces of a two-POTS stimulus for a 3-D overestimation phenomenon to occur.

These results are not consistent with our prediction from the back-surface-bias hypothesis that the number of perceived elements on a front-white/back-black two-POTS stimulus is likely to be larger than that on a front-black/back-white two-POTS stimulus; we did not find any difference in the number of perceived elements between the two types of two-POTS stimuli. This inconsistency, however, does not necessarily show the invalidity of the hypothesis if our manipulation of the luminance of the surfaces was not successful in testing the hypothesis. Thus, we need further investigation to test the hypothesis; we examine the hypothesis again in Experiment 2 using other sets of stimuli.

Even if our manipulation was not effective, the back-surface-bias hypothesis has difficulty explaining the whole set of results of this experiment. According to the hypothesis, if the back surfaces of each of three two-POTS stimuli were treated as a background surface, then the 3-D overestimation phenomenon should occur for each of three two-POTS stimuli. However, when the disparity of the two-POTS stimulus was large, the phenomenon was observed only for a black-white two-POTS stimulus but not for a front-white/back-black and a front-black/back-white two-POTS stimulus; and when the disparity was small, the phenomenon was observed for each two-POTS stimulus. To explain why the phenomenon disappeared when the disparity is large only for the black-white two-POTS stimuli, we need another assumption. We discuss this issue later in General Discussion.

The fact that the disparity had no effect on the number of perceived elements for a black-white two-POTS stimulus is apparently at odds with the result of Experiment 2 in Aida et al. (2015) [14]; they reported that the perceived number of elements for a two-POTS stimulus depended on its disparity size. In their study, the degree of a 3-D overestimation phenomenon for the two-POTS stimulus with 4.0 arcmin was smaller than that with 8.0 arcmin or 12.0 arcmin, while no difference between with 8.0 arcmin and with 12.0 arcmin. In our study, there was no difference in the number of perceived elements between when the elements had 6.8 arcmin and when they had 12.7 arcmin. The apparently different results are explained by assuming that the perceived elements would effectively increase when the disparity of the stimulus is somewhere between 4.0 and 6.8 arcmin.

Third, we analyzed a two-way repeated measures ANOVA (3 two-POTS stimuli × 2 binocular disparities) on the JND. The analysis showed that the main effect of the stimulus, *F* (*2*, *12*) = 3.81, *p* = 0.052, that of the binocular disparity, *F* (1, 6) = 0.23, *p* = 0.65, and their interaction, *F* (2, 12) = 0.32, *p* = 0.73, were not statistically significant. As can be seen in Table 1, the mean JNDs were similar to each other among the three two-POTS stimuli and the two binocular disparities, which is consistent with the result of ANOVA. The similar mean JNDs indicate that as a group, the task difficulty was almost the same among the stimulus conditions.

**Table 1:** Results from Experiment 1. Mean JND and its standard deviation for each two-POTS stimulus were calculated for each disparity condition.

	Disparity			
	6.8 arcmin		12.7 arcmin	
Two-POTS stimulus	Mean SD		Mean SD	
Black-white	24.45	14.69	25.98	10.10
Front-black/back-white	30.91	9.81	27.89	13.44
Front-white/back-black	17.58	11.41	24.08	12.18

## Experiment 2

**Fig 5:**
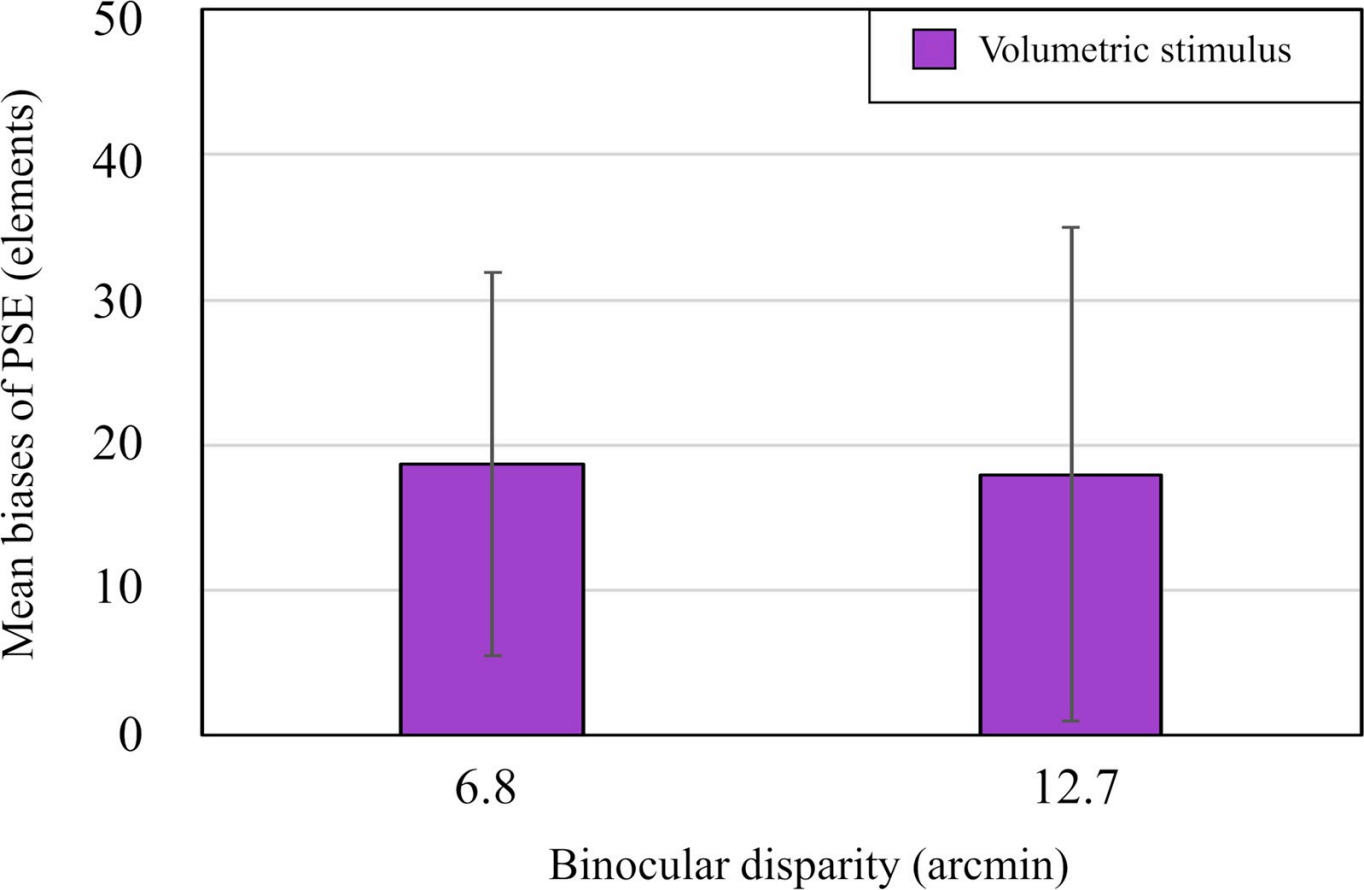
Results from Experiment 2. Mean biases of PSE for volumetric stimuli. The vertical and horizontal axes represent the mean biases of PSE and binocular disparity, respectively. Error bars indicate 95% confidence interval.

To examine the back-surface-bias hypothesis, we manipulated the likelihood that the surface was considered to be background by using a 3-D stimulus that had elements with five different disparities. Our logic for the manipulation was based on the results of previous studies that five or more overlapping “surfaces” are difficult to be perceived as surfaces when inter-surface disparity measures about 3.8 arcmin or below (see Fig 5 in Tsirlin, et al., 2008 [22]). Thus, if the 3-D stimulus would not appear as a five-POTS stimulus but as a volume, the numerosity overestimation would not occur.

### Method

#### Stimuli

The 3-D standards stimuli were volumetric stimuli (see Fig 1B). The total number of elements on the 3-D standard was 300, among which every 60 elements had five different binocular disparities (see General methods). The elements with the same disparity consisted of 30 white and 30 black elements. The numbers of simultaneously presented 2-D stimulus elements varied at five levels with 300 set as the baseline. The initial values and ranges of the step sizes for the five levels for the 2-D comparison were varied among different observer as in Experiment 1. The initial values ranged from 176 to 196, and the step sizes ranged from 52 to 62.

#### Procedure

The experiment comprised two blocks: in one block, the outmost elements of the 3-D standard stimulus had 6.8 arcmin disparity, and in the other, the outmost elements had 12.7 arcmin disparity. Half of 14 observers were assigned to the smaller-disparity condition block and the other half to the larger-disparity condition block. In each block, the location of the 3-D standard (left or right) and the number of elements on the 2-D comparison were randomly selected with five repetitions. Consequently, each observer was presented with stimuli for a total of 50 times (2 locations of the standard ×5 numbers of elements on the comparison × 5 repetitions).

In each trial, observers were asked whether the 3-D standard (volumetric) stimulus was seen as overlapping surfaces or not, and if any, how many surfaces were seen, before selecting one of the two stimuli (2-D comparison and 3-D standard) appeared to contain more elements than the other. This “surface” task was performed to examine whether observers observed the volumetric stimulus as a five-POTS or not.

### Results and discussion

Before examining the bias of the PSE, we examined whether the 3-D standard appeared as a five-POTS or not. For each disparity condition, four out of seven observers reported that they did not see any surfaces, and the remaining three reported that they observed three surfaces. These results indicate that all the14 observers were not able to perceive five-POTS, being consistent with the previous data [22]. This result, however, only partially supported our expectation that a background surface would not be perceived in our “volumetric” stimuli. We discuss this issue later in this section.

Then, we conducted a t-test on the bias of the PSE. The analysis showed that the bias, *t* (12) = 0.07, *p* = 0.95, was not statistically significant between the two disparity conditions. Fig 5 shows the mean biases separately for each disparity with the error bars indicating 95% CI. As can be seen in Fig 5, the mean biases are nearly the same between the two disparity conditions, and the 95% CI in each disparity condition overlapped, being consistent with the result of the t-test.

Next, we analyzed the 95% CI for the mean bias of each disparity condition as an index of the overestimation phenomenon as in Experiment 1. As can be seen in Fig 5, the lower limit is larger than zero for each disparity condition (95% CI of 18.7 ± 13.2 when the disparity of the outmost elements was 6.8 arcmin and that of 18.0 ± 17.0 when it was 12.7 arcmin.) The fact that the minimum value of 95% CI was smaller than zero for the volumetric stimulus suggests that the number of elements on the stimuli was overestimated.

**Fig 6:**
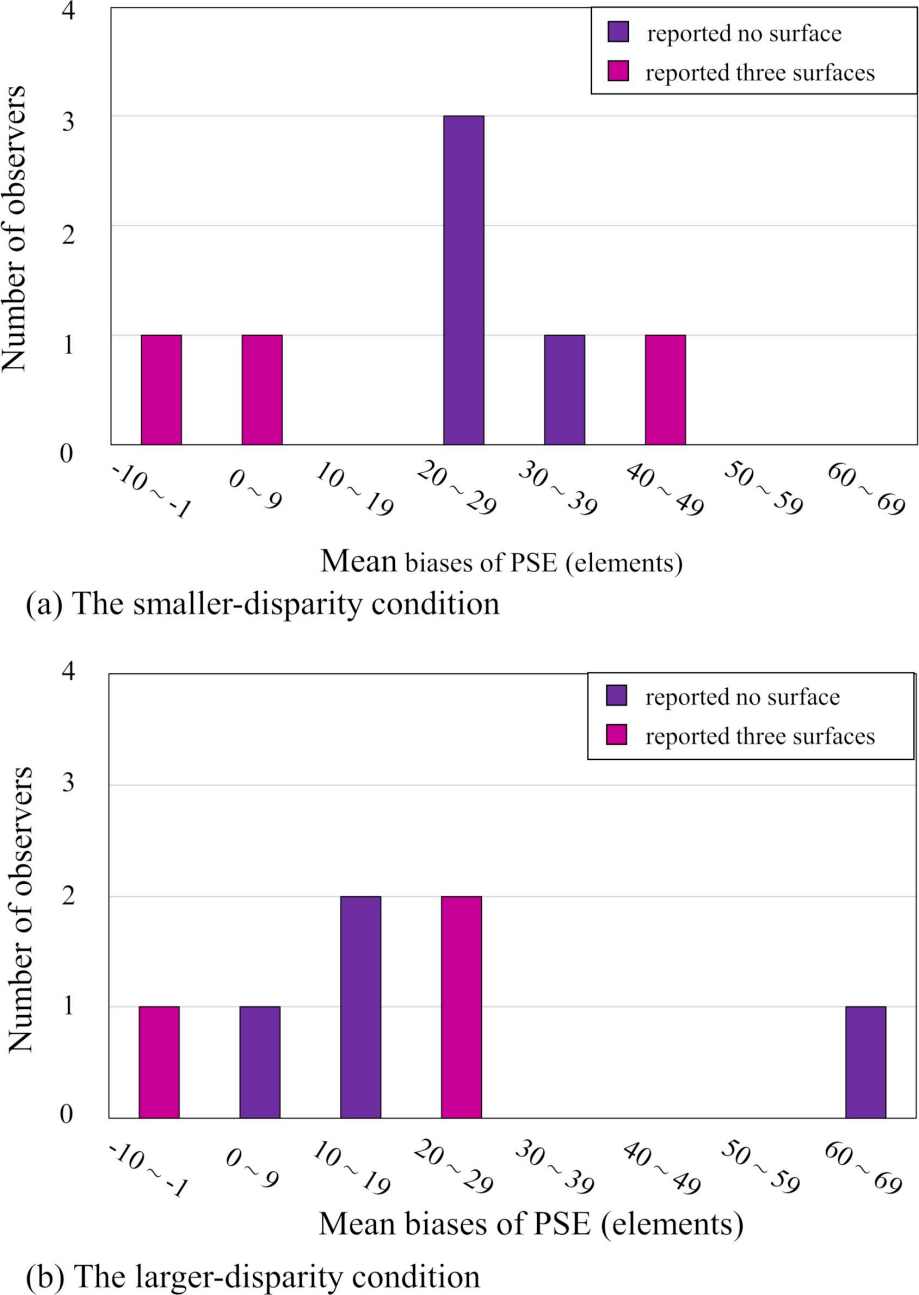
The number of observers as a function of PSE bias. The number of observers as a function of PSE bias for the smaller-disparity condition (a) and the larger-disparity condition (b). The vertical and horizontal axes represent the number of observers and the mean biases of PSE, respectively.

Further, we examined whether the perception of a background surface had an effect on the perceived number of 3-D stimulus elements. If the observers' report that three surfaces are perceived means that the observers view a background surface and if the back-surface-bias hypothesis is valid, then it is expected that a 3-D overestimation phenomenon occurs only when the three surfaces are reported in this experiment. To examine the expectation, we plotted the relation between the PSE bias and the perception as to whether the surface was perceived or not in Fig 6; the number of the observers was depicted as a function of the PSE bias with the parameter of the perception of surface, separately for the smaller-disparity (Fig 6A) and larger-disparity conditions (Fig 6B). As can be seen in Fig 6, the PSE biases of six observers out of seven are each larger than zero in each disparity condition, showing that the overestimation occurred irrespective of whether a background surface was observed or not. This result indicates that the back-surface hypothesis cannot explain all the overestimation phenomena obtained in this experiment.

One might think that the result that a 3-D overestimation phenomenon was observed in this experiment is apparently at odds with the result of Experiment 2 in Bell et al. (2015) [15]; they reported that the phenomenon was not observed for a cylindrical volumetric stimulus, where there was no background surface. Because there were several differences in the stimulus presentation and the stimulus properties between the two experiments, it is difficult to make it clear which factor played a role in the difference between the two results. However, we prefer to interpret the difference of the results as due to the difference in the size of areas containing elements between the two experiments. In Bell et al. [15], the size of areas containing elements was 5.6° arcdeg, while in this experiment, the size was 8.6° × 8.6° arcdeg. As Matsuda, Shimono, & Aida (2017) [16] reported, the amount of perceived number of 3-D stimulus elements decreased as the size of areas decreased. If this is the case, the fact that a 3-D overestimation phenomenon was not observed in Bell et al., can be, at least partially, due to the areal size they used.

Third, we conducted a t-test on the JND. The analysis showed that the JND was not significantly different between the two disparity conditions, *t* (12) = 0.53. *p* = 0.61. The mean JND averaged over seven observers was 27.48 with a standard deviation (SD) of 19.05 in the smaller-disparity, and that was 22.91 with a standard deviation of 12.72, suggesting that the task difficulty was almost the same between the two disparity conditions. Note that the mean JNDs in this experiment were also consistent with those reported in Experiment 1. We discuss the similarity in the JND measured in this study later in Experiment 3.

## Experiment 3

We evaluated the occlusion hypothesis that assumes that the elements perceived on a front surface can occlude elements behind [14] and the number of the occluded elements is added to the number of the physical elements, resulting in a 3-D overestimation phenomenon. According to the hypothesis, a process taking into account the number of “occluded” elements in numerical estimation may not operate effectively when a 3-D stimulus has no back surface as a 2-D stimulus.

In this experiment, we manipulated the likelihood that possibly occluded elements are added, by using a stepwise 3-D stimulus (Fig 1C). We expected that when two flat surfaces in the 3-D stimulus are not overlapped, the process taking into account for the occluded elements is less likely to operate as for a 2-D stimulus. Consequently, the overestimation of elements would not occur for the stepwise 3-D stimulus.

### Method

#### Stimuli

The 3-D standards stimuli were one black-white two-POTS and four different stepwise stimuli. The two-POTS stimulus was the same as that used in Experiment 1. The stepwise stimulus had two flat surfaces that had the same total disparity as the two-POTS stimulus but its two surfaces were not overlapped; the left or right of the two flat surfaces appeared in front of the other half, or the upper or lower half of the two flat surfaces appeared in front of the other half (see Fig 1C). The total numbers of elements on the two-POTS stimulus and stepwise stimulus were the same (300). The number of elements on each half surface of the stepwise stimulus was 150, of which 50% were white and 50% black. The numbers of simultaneously presented 2-D stimulus elements varied at five levels with 300 set as the baseline as in Experiments 1 and 2. The initial values and ranges of the step sizes for the five levels for the 2-D comparison were varied among different observer as in Experiments 1–3. The initial values ranged from 180 to 252, and the step sizes ranged from 24 to 60.

#### Procedure

The experiment comprised two blocks: in one block, the two surfaces of the 3-D standard stimulus had 6.8 arcmin disparity, and in the other, they had 12.7 arcmin disparity as in Experiment 2. Half of 14 observers were assigned to the block for the smaller-disparity condition block and the other half to the larger-disparity condition block as in Experiment 2. In each block, one of the five 3-D standards (one two-POTS and four stepwise stimuli), location of the standard (left or right), and the number of elements on the comparison were randomly selected with five repetitions. Consequently, each observer was presented with stimuli for a total of 250 times (5 standards x 2 locations of the standard ×5 numbers of elements on the comparison × 5 repetitions).

### Results and discussion

First, we conducted a two-way mixed-design ANOVA (5 standards stimuli × 2 binocular disparities) performed for the bias of the PSE, with binocular disparity as a between-observers variable and with standard stimulus as a within-observer variable. The analysis showed that the main effects of the standard, *F* (4, 48) = 4.08, *p* = 0.01, and binocular disparity, *F* (1, 12) = 4.82, *p* = 0.05, were statistically significant, while their interaction, *F* (4, 48) = 1.00, *p* = 0.42, was not.

**Fig 7:**
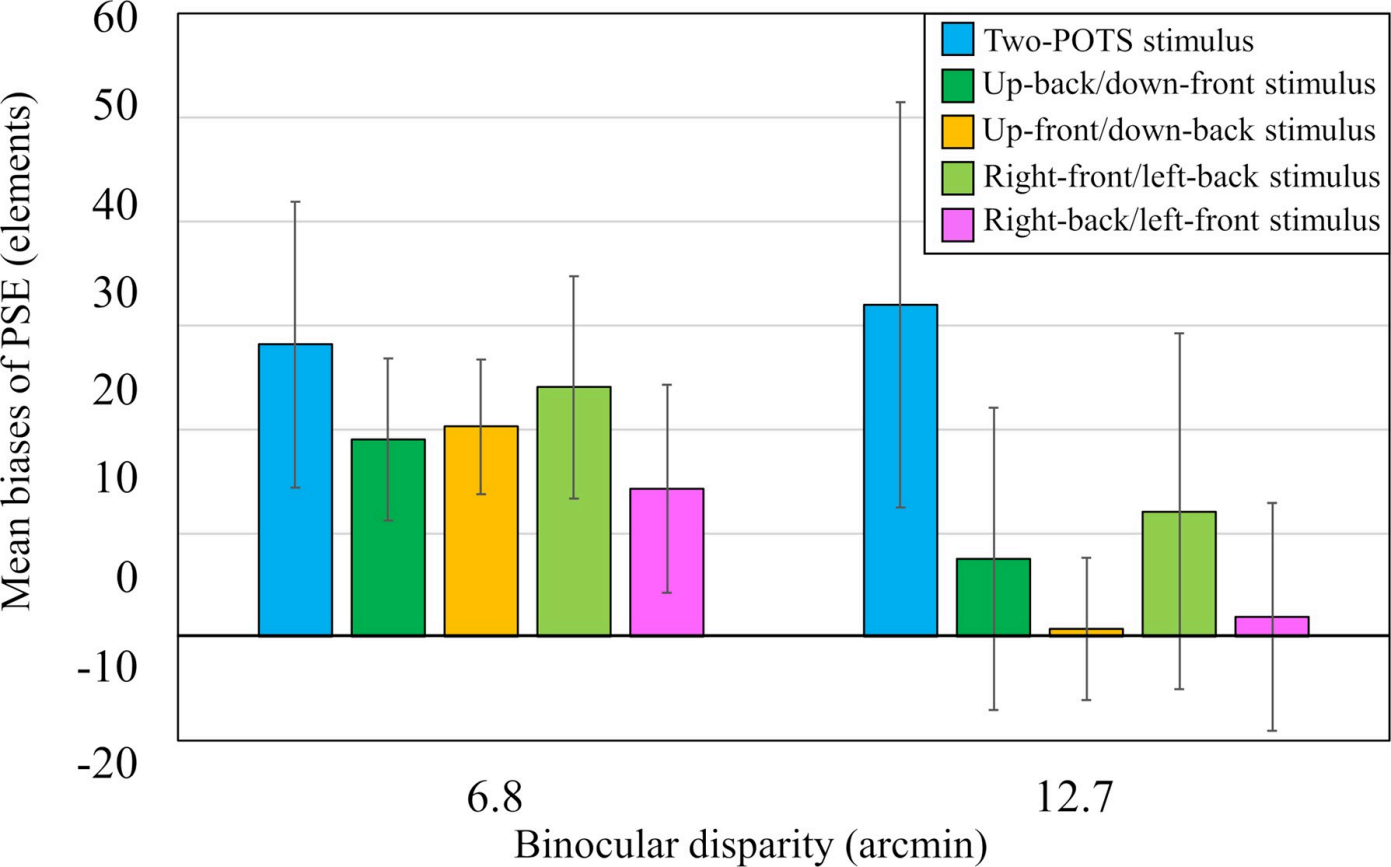
Results from Experiment 3. Mean biases of PSE for each of the five different 3-D standard stimuli. The vertical and horizontal axes represent the mean biases of PSE and binocular disparity, respectively. Error bars indicate 95% confidence interval.

The statistical results can be seen in Fig 7, in which the mean biases are depicted for each standard stimulus with the binocular disparity as a parameter with the error bars indicating 95% CI. As can be seen in Fig 7, the mean biases of the four stepwise stimuli were each larger for the smaller-disparity condition than for the larger-disparity condition except for the two-POTS stimulus, whose mean bias was almost the same between the two disparity conditions.

Next, we analyzed the 95% CI for the mean bias as an index of the overestimation phenomenon as in Experiments 1 and 2. As can be shown in Fig 7, the limit was larger than zero in each of the 3-D stimuli for the smaller-disparity condition while it was larger than zero only for the two-POTS stimulus but smaller than zero in each of the four stepwise stimuli for the larger-disparity condition. The 95% CIs for the two-POTS, up-back/down-front, up-front/down-back, right-front/left-back, and right-back/left-front stimuli were 28.2 ± 13.8, 19.0 ± 7.8, 20.3 ± 6.5, 24.1 ± 10.7, and 14.3 ± 10.0, respectively, for the smaller-disparity condition; and 32.0 ± 19.5, 7.5 ± 14.5, 0.8 ± 6.8, 12.1 ± 17.1, and 1.9 ± 11.0, respectively, for the larger-disparity condition. The results indicated that the numerical overestimation of the 3-D stimulus was observed for the smaller disparity condition while it was not for the larger-disparity condition. The result for the smaller disparity condition contradicts the prediction of the occlusion hypothesis while that for the larger-disparity condition is consistent with it.

One might argue, however, that the occlusion hypothesis can explain the result of the smaller-disparity condition by assuming that the visual system takes into account for the number of hidden elements, which can be occluded by the elements on a front surface in a stepwise stimulus when the disparity between two surfaces is relatively small. If this is the case, the number of perceived elements in the front surface of the stepwise stimulus can be overestimated, compared to that of a single flat surface or a 2-D stimulus. In Experiment 4, we explored this prediction.

Third, we conducted a two-way mixed-design ANOVA (5 3-D stimuli × 2 binocular disparities) performed for the JND, with binocular disparity as a between-observers variable and with 3-D stimulus as a within-observer variable. The analysis showed that the main effect of the 3-D stimulus, *F* (4, 48) = 1.98, *p* = 0.11, that of binocular disparity, *F* (1, 12) = 2.71, *p* = 0.13, and their interaction, *F* (4, 48) = 0.78, *p* = 0.54, were not statistically significant. As can be seen in Table 2, the mean JNDs were similar to each other among the five 3-D stimuli and the two binocular disparities, which is consistent with the result of ANOVA. The results indicate that the task difficulty was similar among the stimulus conditions as a group. The results also show that the mean JNDs in this experiment were consistent with those in Experiments 1 and 2.

**Table 2:** Results from Experiment 3. Mean JND and its standard deviation for each of the five different 3-D standard stimuli were calculated for each disparity condition.

	Disparity			
	6.8 arcmin		12.7 arcmin	
3-D stimulus	Mean SD		Mean SD	
Two-POTS	26.16	11.89	27.74	15.65
Up-back/down-front	24.96	7.42	21.43	5.64
Up-front/down-back	21.54	11.18	17.78	8.71
Right-front/left-back	32.90	12.38	21.83	8.75
Right-back/left-front	23.11	9.86	17.01	4.93

It may be interesting to compare the JND of the 3-D stimulus obtained in Experiments 1–3 and that of the 2-D stimulus obtained in the previous studies. Because the number of elements on the standard stimulus is known to affect the JND or Weber fraction (JND expressed as a fraction of the number of the elements on the standard stimulus) for the 2-D stimulus (e.g., [23, 24]), we use the data obtained when the number of elements was relatively close to that used in this study in the following discussion. The JND averaged over 18 mean JNDs of the 3-D standard in this study was 24.21, where the number of elements on the standard stimulus was 300. The corresponding Weber fraction was 0.08. The value is very well comparable with those obtained in the previous studies (e.g., [23, 24, 25, 26]). For example, Krueger (1984) [26] reported that the JND for the standard containing 400 elements was 32.3, and thus, Weber fraction was 0.08. From Fig 2A in Anobile et al. (2015) [23], we can read that Weber fraction for the standard containing 300 elements was around 0.08. The fact that Weber fraction for the 3-D stimulus agrees well with that for the 2-D stimulus suggests that task difficulty to discriminate the number of elements on a 2-D stimulus and that on 3-D stimulus is virtually the same as that to discriminate between the number of elements on a 2-D stimulus.

## Experiment 4

To examine the occlusion hypothesis further, we compared the number of perceived elements on the front or back surface of a 3-D stepwise stimulus to that on a single flat surface (2-D stimulus). If the 3-D overestimation phenomenon observed in Experiment 3 is referable to the possible occlusion of elements on the front surface of the stepwise stimulus, the phenomenon would occur for the elements on the front surface but not for those on the back surface.

### Method

#### Stimuli

**Fig 8:**
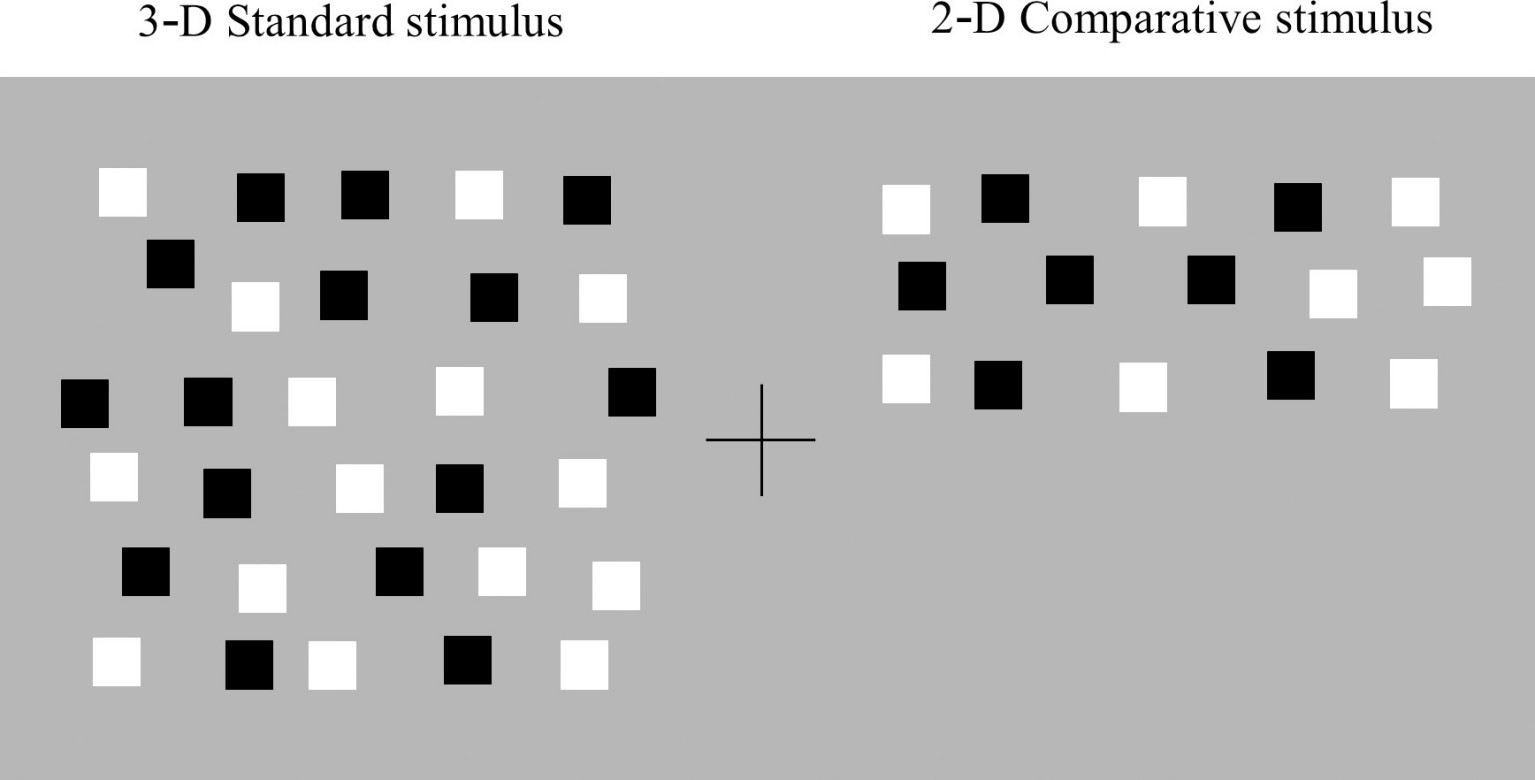
Schematic front view of stimulus used in Experiment 4. The width and the height of the 2-D stimulus were the same as and half of the 3-D stimulus, respectively.

The 3-D stimuli comprised two stepwise (up-back/down-front and up-front/down-back) stimuli, which were the same as used in Experiment 3. Thus, the size (width and height) of the 3-D stimuli was the same as that used in Experiment 3. The width and the height of the 2-D stimuli were the same as and half of that used in Experiment 3, respectively. Fig 8 shows the schematic front view of the stimulus elements displayed on the monitor. The 2-D stimulus was presented on the monitor plane so that its top line was aligned with that of the 3-D stimulus (Fig 8) or its bottom line. For the up-back/down-front stepwise (3-D) stimulus, the upper and lower surface appeared to be behind and in front of the 2-D stimulus, respectively; and for the up-front/down-back stepwise (3-D) stimuli, the upper and lower surfaces appeared to be in front of or behind the 2-D stimulus, respectively. The number of 3-D stimulus elements in the upper and lower surfaces was each 150. The numbers of 2-D stimulus elements simultaneously presented varied at five levels with 150 set as the baseline. The initial values and ranges of the step sizes for the five levels for the 2-D comparison were varied among different observer as in Experiments 1–3. The initial values ranged from 70 to 110, and the step sizes ranged from 20 to 40.

#### Procedure

The experiment comprised two blocks: in one block, the two surfaces of the 3-D standard stimulus had 6.8 arcmin disparity, and in the other, they had 12.7 arcmin disparity as in Experiments 2 and 3. Half of 14 observers were assigned to the smaller-disparity condition and the other half to the larger-disparity condition. Observers were asked to perform the same two-alternative forced-choice task as in all the other three experiments but asked to compare the number of the 2-D stimulus and that of the front or back surface of the stepwise stimuli. Thus, there were four estimation conditions: two for the up-back/down-front stepwise stimulus (up-back estimation and down-front estimation) and the other two for the up-front/down-back stepwise stimulus (up-front estimation and down-back estimation). In each disparity condition, the location of the 3-D stimulus (left or right) and the number of elements on the 2-D comparison were randomly selected with five repetitions. Consequently, each observer was presented with stimuli for a total of 50 times (2 locations of the standard ×5 numbers of elements on the comparison × 5 repetitions).

### Results and discussion

**Fig 9:**
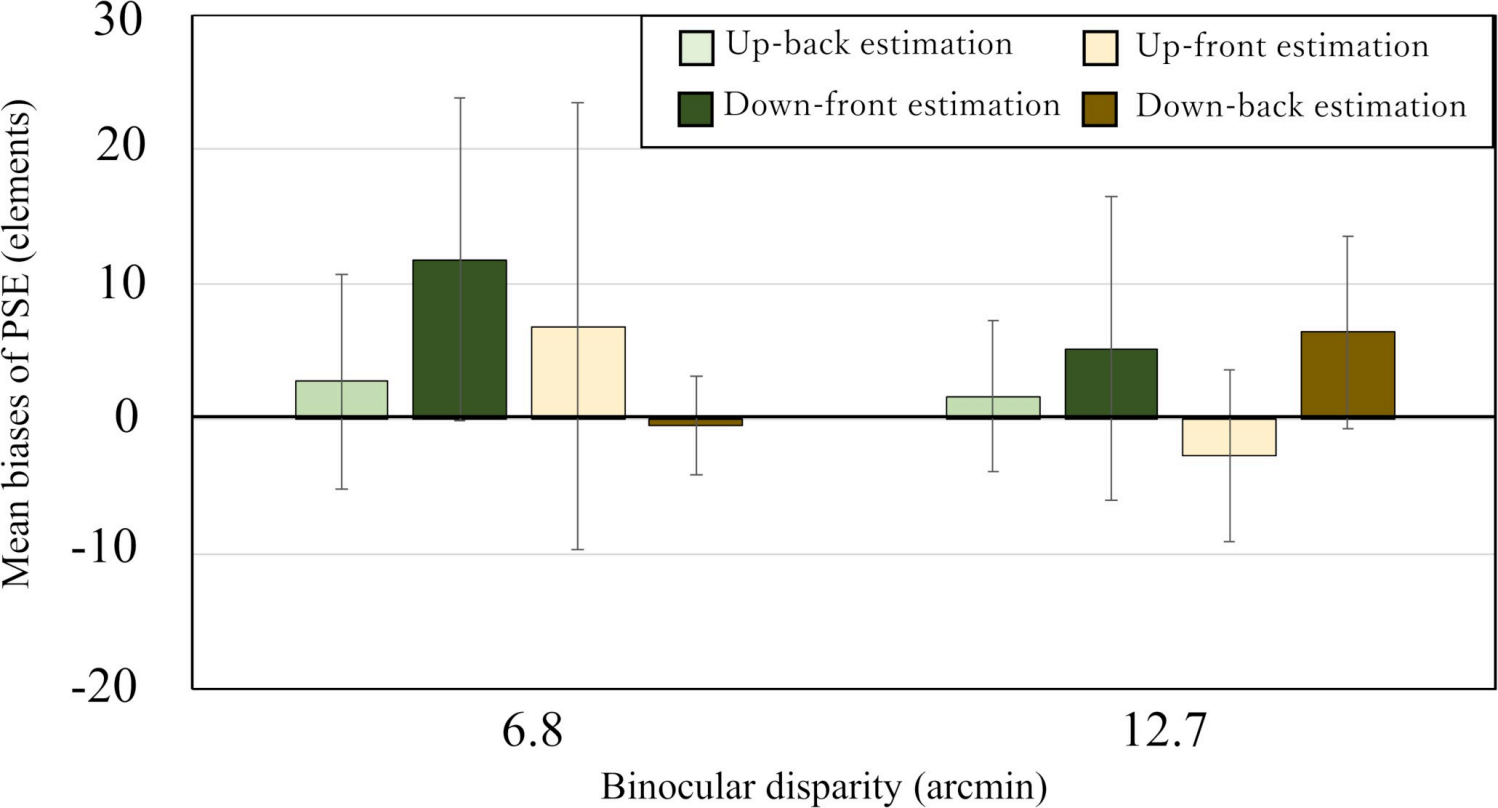
Results from Experiment 4. Mean biases of PSE for the four estimation conditions (see text for details). The vertical and horizontal axes represent the mean biases of PSE and binocular disparity, respectively. Error bars indicate the 95% confidence interval.

First, we conducted a two-way mixed-design ANOVA (4 estimation conditions × 2 binocular disparities) on the biases of the PSE with binocular disparity as a between-observers variable and with estimation condition as a within-observer variable. The analysis showed that the main effects of the estimation condition, *F* (3, 36) = 0.88, *p* = 0.46, and binocular disparity, *F* (1, 12) = 0.40, *p* = 0.54, and their interaction, *F* (3, 36) = 1.24, *p* = 0.31, were not statistically significant. Fig 9 shows the mean biases of PSE for each estimation condition separately with the binocular disparity as a parameter with the error bars indicating 95% CI. As can be seen in Fig 9, although the mean biases are not necessarily the same among all conditions, but the 95% CIs in all the conditions overlapped to each other. This result is consistent with that of ANOVA.

Next, we examined whether the lower limit of the 95% CI of the PSE bias in each estimate condition was larger than zero or not as in Experiments 1 to 3. As seen in Fig 9, the minimum limits of the 95% CIs of all the estimation conditions are smaller than zero in each disparity condition; when the binocular disparity was small, the 95% CIs of the up-back, down-front, up-front, and down-back estimation conditions were 2.8 ± 8.0, 11.8 ± 11.9, 6.9 ± 16.6, and −0.5 ± 3.7, respectively, and when the binocular disparity was large, the 95% CIs of the up-back, down-front, up-front, and down-back estimation conditions were 1.7 ± 5.6, 5.2 ± 11.3, −2.7 ± 6.3, and 6.5 ± 7.1, respectively. These results indicate that no overestimation of the number of 3-D stimulus elements occurred in any of the conditions. Thus, the results contradict the prediction of the occlusion hypothesis, indicating that it is incapable of explaining the occurrence of numerical overestimation observed in Experiment 3.

The finding that the overestimation phenomenon was observed in the smaller-disparity condition in Experiment 3 but not in this experiment suggests that the phenomenon cannot be due to the simple summation of the perceived number of elements on the front and back surfaces. This suggestion is consistent with the finding in Aida et al. (2015). They used a two-POTS stimulus and measured the perceived number of elements on its front and back surfaces separately and together They found that when the stimulus contained 300 elements, the summation between the perceived number of the front and back surfaces, measured separately, was two times larger than that measured together (see Figs 4 and 5 in Aida et al., 2015). As discussed in Aida et al., to explain these findings, “we need to assume that the visual system sums the perceived numbers for a front surface and back surface nonlinearly” (p. 13) in judging the number of perceived elements in a stepwise 3-D stimulus.

Third, we conducted a two-way mixed-design ANOVA (4 estimation conditions × 2 binocular disparities) on the JND with binocular disparity as a between-observers variable and with estimation condition as a within-observer variable. The analysis showed that the main effect of the standard, *F* (3, 36) = 0.27, *p* = 0.85, that of binocular disparity, *F* (1, 12) = 0.001, *p* = 0.98, and their interaction, *F* (3, 36) = 1.30, *p* = 0.29, were not statistically significant. As can be seen in Table 3, the mean JNDs were similar to each other among the four estimation conditions and the two binocular disparities, which is consistent with the result of ANOVA. The results indicate that the task difficulty was similar among the stimulus conditions as a group.

**Table 3:** Results from Experiment 4. Mean JND and its standard deviation for each estimation condition were calculated for each disparity condition.

	Disparity			
	6.8 arcmin		12.7 arcmin	
Estimation condition	Mean SD		Mean SD	
Up-back estimation	14.24	7.50	11.84	7.80
Down-front estimation	11.33	4.29	14.77	4.09
Up-front estimation	13.34	4.07	10.96	6.17
Down-back estimation	13.20	6.23	14.24	8.04

Furthermore, the averaged JND over eight mean JNDs in this experiment and its corresponding Weber fraction were 12.62 and 0.08, respectively. The value of Weber fraction in this experiment very well corresponds with that obtained in Experiments 1–3 as well as that for a 2-D stimulus reported in the previous studies (e.g., [23, 24, 25, 26]). This correspondence is consistent with the idea discussed in Experiment 4 that task difficulty in discriminating the number of elements in a 2-D space is similar to that in discriminating the number of elements in a 2-D space and that of elements in a 3-D space.

## General discussion

In this study, four experiments (Experiments 1–4) were conducted to examine the predictions of two hypotheses explaining the numerical overestimation of the number of elements in a 3-D stimulus that depicted parallel-overlapping-transparent-stereoscopic-surfaces (POTS). In Experiments 1 and 2, we examined the prediction of the back-surface-bias hypothesis that the overestimation would not occur when elements seen behind in a POTS stimulus are less likely to be regarded as an opaque background surface [14, 17, 19]. In Experiment 1, our manipulation of the possibility of being considered as an opaque background surface had no effects on the amount of the numerosity estimation. In Experiment 2, the overestimation phenomenon was observed regardless of whether a five-POTS stimulus was perceived to contain background surfaces, or not. In Experiments 3 and 4, we examined the prediction of the occlusion hypothesis that the overestimation would not occur when a front surface does not overlap a back surface in a POTS stimulus [14, 17]. In Experiment 3, the overestimation was observed for a 3-D stimulus that had two non-overlapping surfaces, when the disparity between the two surfaces was relatively small but not observed when the disparity was relatively large. In Experiment 4, the overestimation of the number of 3-D stimuli elements was not observed in each of the two surfaces. Taken together, either the back-surface-bias or the occlusion hypothesis has difficulty in explaining the results by itself.

Our results can be summarized as follows. When binocular disparity of 3-D stimulus elements was relatively small, the numerosity overestimation occurred for the 3-D stimuli we used: a two-POTS stimulus, where two stereo-surfaces were overlapped parallelly; a volumetric stimulus, where constituent elements in a five POTS stimulus were not perceived as five parallel and overlapping surfaces, and a stepwise stimulus, where two surfaces in depth were not overlapped. When binocular disparity was relatively large, the overestimation diminished for a two-POTS stimulus, where the averaged luminance differed between the two surfaces, and for a stepwise stimulus, but occurred for a two-POTS stimulus, where the averaged luminance of the two surfaces was the same, and for the volumetric stimulus. These results indicate that it depends on the disparity size of 3-D stimulus elements and depth structure of the stimulus for a 3-D overestimation phenomenon to occur.

To account for the 3-D overestimation phenomenon, we propose a new hypothesis that processing disparities of elements in a 3-D stimulus interferes with the numerosity estimation of the elements. According to this hypothesis, the interference results in an overestimation of the elements in the 3-D stimulus. The hypothesis can explain the results that the disparity size of 3-D stimulus elements and its depth structure affected the overestimation phenomenon by assuming that the degree of interference depends on these two factors. For example, it can explain the results that the disparity range for the overestimation to occur differed among different 3-D stimuli by assuming that the degree of the interference is larger when 3-D elements are overlapped in depth than when they are not. Furthermore, the hypothesis explains the fact that the phenomenon is less likely to occur when the scope of elements is relatively small [15, 16] by assuming that the degree of the interference becomes less as the scope becomes small. However, we do not have a clear explanation why the disparity range depended on whether the averaged luminance of a front surface is the same as or different from that of a back surface of the two-POTS stimulus (see Fig 4).

The disparity-processing-interference hypothesis is qualitatively consistent with the previous finding on density judgment [27]. Sun and Baker (2018) [27] reported that the perceived density of a central circular flat surface is affected when a 3-D stimulus containing random-dots elements surrounds the circular surface; the amount of simultaneous density contrast of the central circular surface reduces as the disparity of 3-D stimulus elements increases. This reduction is observed irrespective of whether the 3-D surrounding stimulus is a two-POTS stimulus or a volumetric stimulus. These findings can be understood if we assume that (a) processing the disparity of the elements surrounding the central surface interferes with the density judgments and (b) the degree of inference increases as the disparity increases to reduce the amount of simultaneous density contrast.

In this study, we also compared the task difficulty using an index of just noticeable difference (JND) or Weber fraction (JND per the number of elements on the standard stimulus) in four experiments. Experiments 1–4 showed that the task difficulty or discrimination sensitivity between 2-D and 3-D elements was almost the same among the stimulus conditions used. The task difficulty found in this study was also the same as that in the previous studies, in which discrimination sensitivity was measured between 2-D elements [23, 24, 25, 26]. Thus, the three-dimensionality of the elements did not affect the sensitivity to numerosity, while it did the perceived numerosity. To explain the result by assuming that disparity processing of 3-D elements interferes with their numerosity estimation, we may need to assume that the interference affects perceived numerosity but not discrimination sensitivity.

Finally, in the present study, we found that it depended on disparity size and depth structure of a stereoscopic 3-D stimulus whether numerical overestimation of elements in the stimulus occurred or not; the overestimation is observed irrespective of perceived structure of the 3-D stimulus used when the binocular disparity was relatively small, while it depended on the depth structure (e.g., overlapping of stereo-surfaces and difference of the average luminance between stereo-surfaces) when the binocular disparity was relatively large. These findings are not consistent with either of the two previously proposed hypotheses [14, 17, 19]. We proposed a hypothesis that holds that processing the disparity of 3-D stimulus elements puts a load on the numerosity estimation to explain the findings. The hypothesis, however, does not necessarily describe the whole set of findings in this study, suggesting that several factors may factor in the numerosity overestimation phenomenon.

## Supporting information

S1 Rawdata (XLSX)
